# The influence of gender on COVID-19 infections and mortality in Germany: Insights from age- and gender-specific modeling of contact rates, infections, and deaths in the early phase of the pandemic

**DOI:** 10.1371/journal.pone.0268119

**Published:** 2022-05-06

**Authors:** Achim Doerre, Gabriele Doblhammer

**Affiliations:** 1 Department of Economics, University of Rostock, Rostock, Germany; 2 Department of Sociology and Demography, University of Rostock, Rostock, Germany; Nanyang Technological University, SINGAPORE

## Abstract

Recent research points towards age- and gender-specific transmission of COVID-19 infections and their outcomes. The effect of gender, however, has been overlooked in past modelling approaches of COVID-19 infections. The aim of our study is to explore how gender-specific contact behavior affects gender-specific COVID-19 infections and deaths. We consider a compartment model to establish short-term forecasts of the COVID-19 epidemic over a time period of 75 days. Compartments are subdivided into different age groups and genders, and estimated contact patterns, based on previous studies, are incorporated to account for age- and gender-specific social behaviour. The model is fitted to real data and used for assessing the effect of hypothetical contact scenarios all starting at a daily level of 10 new infections per million population. On day 75 after the end of the lockdown, infection rates are highest among the young and working-age, but they also have increased among the old. Sex ratios reveal higher infection risks among women than men at working ages; the opposite holds true at old age. Death rates in all age groups are twice as high for men as for women. Small changes in contact rates at working and young ages have a considerable effect on infections and mortality at old age, with elderly men being always at higher risk of infection and mortality. Our results underline the high importance of the non-pharmaceutical mitigation measures (NPMM) in low-infection phases of the pandemic to prevent that an increase in contact rates leads to higher mortality among the elderly, even if easing measures take place among the young. At young and middle ages, women’s contribution to increasing infections is higher due to their higher number of contacts. Gender differences in contact rates may be one pathway that contributes to the spread of the disease and results in gender-specific infection rates and their mortality outcome. To further explore possible pathways, more data on contact behavior and COVID-19 transmission is needed, which includes gender- and socio-demographic information.

## Introduction

Right from the start of the COVID-19 pandemic, the importance of age on COVID-19 contraction and fatality has been recognised (among others, [1–5]), as well as of coresidence patterns [1]. Compartment and agent-based models aiming at projecting the spread of the disease have incorporated age as an important variable of transmission (e.g. [6–10]), in addition to other characteristics such as space [8] or contact patterns [11].

**Fig 1:**
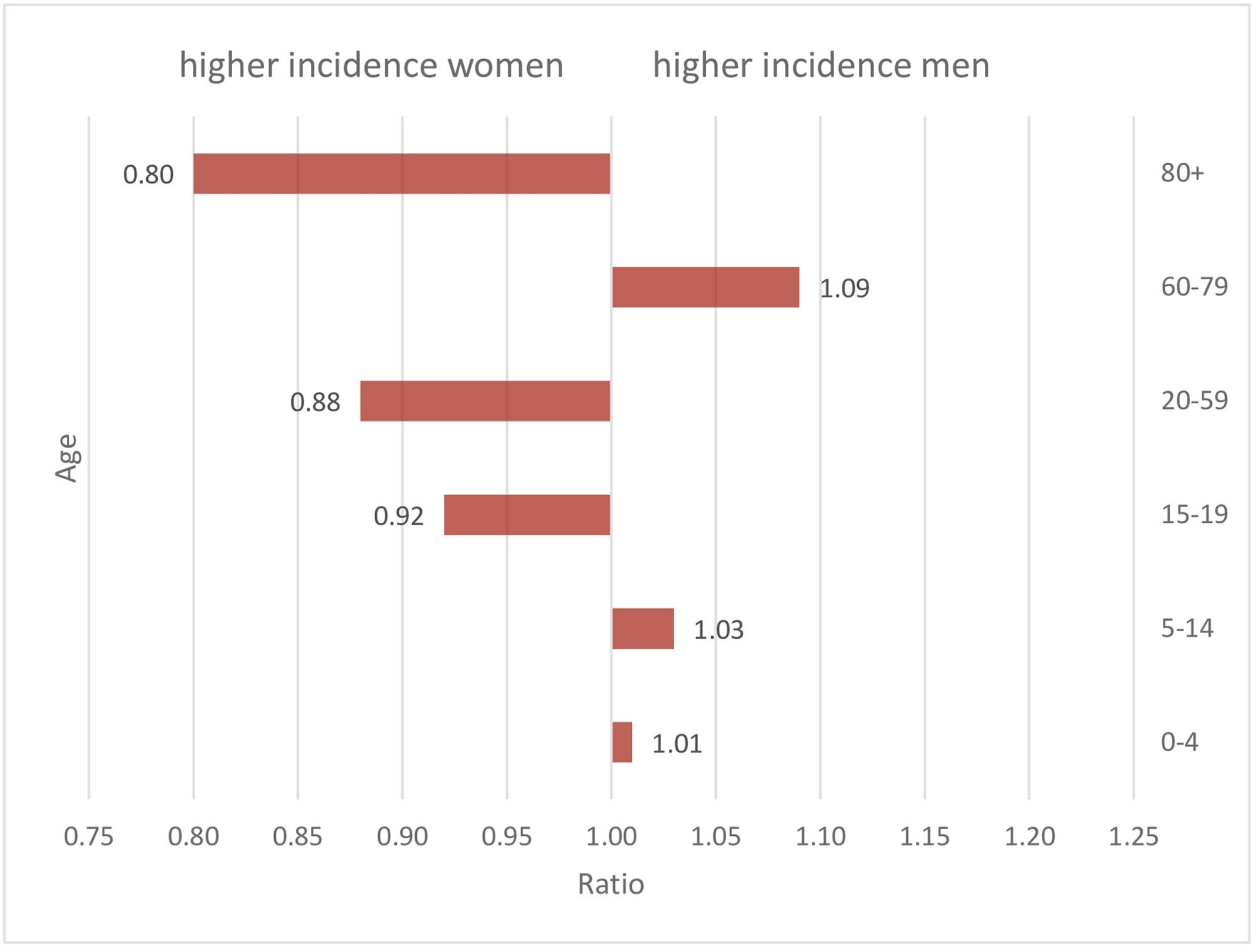
Sex ratios of COVID-19 incidence. Sex ratio (men/women) of COVID-19 incidence through January 5, 2021 by age (data source: Robert Koch Institute Dashboard, authors’ calculations).

An important determinant, which appeared to be largely overlooked in modelling exercises, is sex. While studies generally notice that infection and in particular fatality rates were higher among elderly men than women, the reverse appears to be true for infections at working ages [12]. In Germany, infection rates were higher among women aged 15 to 59 than among men until January 5, 2021, when we started this projection exercise. It was not until the ages of 60 and 70 that men had higher infection rates, which reversed at the age of 80 and above. This pattern remained stable over the early course of the pandemic (Fig 1). To better understand transition pathways, we need to adopt a gender perspective, as has been done extensively in the study of clinical infection outcomes (e.g. [13, 14]).

**Fig 2:**
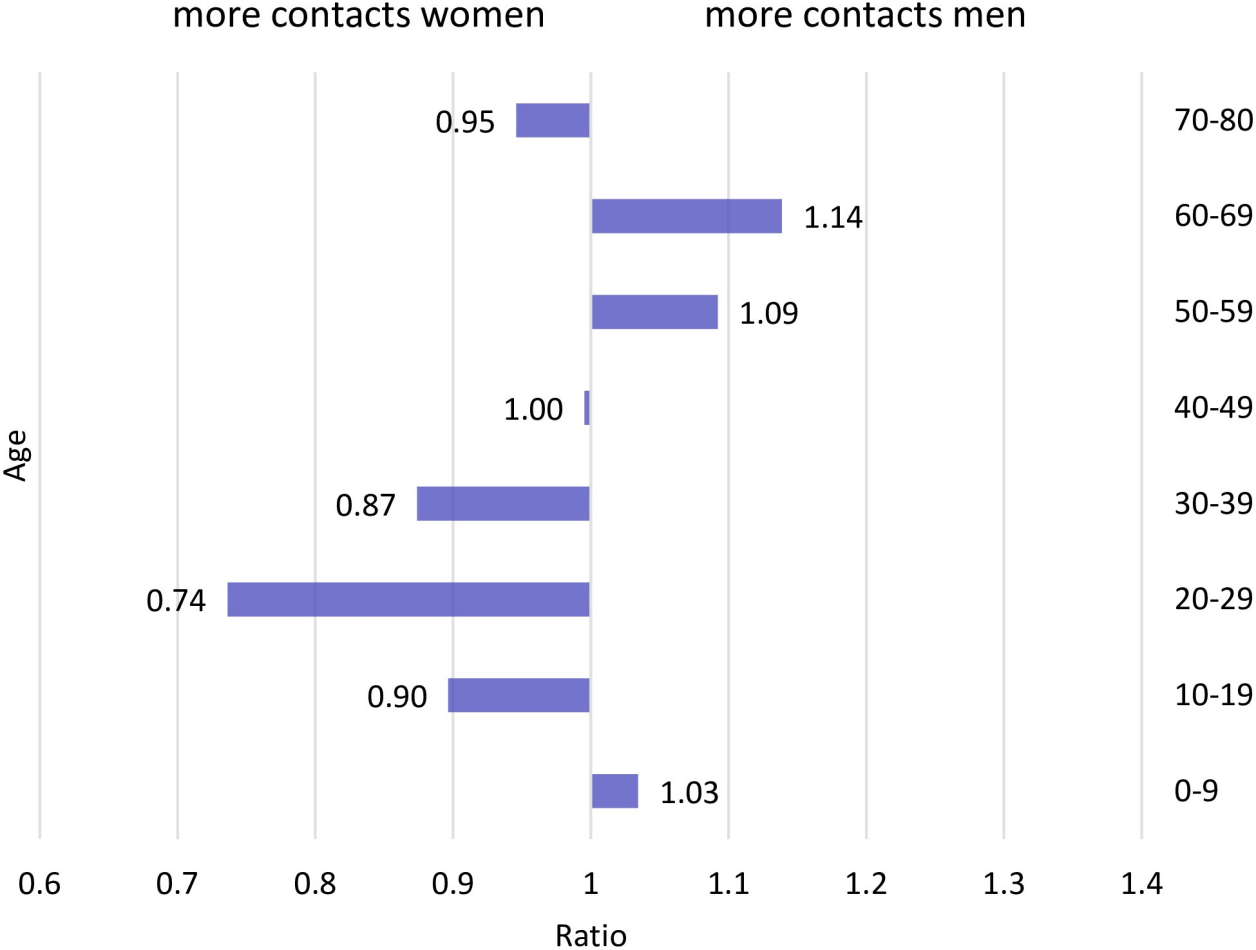
Gender-specific contact rates. Ratio of the average number of contacts among men compared to women, Data Source: [15].

In the following, we will refer to sex when discussing technical details and biological factors, and gender, when referring to social factors. One reason for this difference, in addition to biological factors (see discussion below) lies in gender-specific contact rates. Estimates of contact rates [15] based on the POLYMOD study [16] showed that household, workplace and school structures strongly shape age- and gender-specific contacts made by individuals. Using the contact matrices from the latter study and calculating the ratio of the age-specific number of contacts for men and women (contacts men/contacts women) a clear pattern emerges (Fig 2): among ages 20–39, contacts are between 13%–26% higher among women, while among ages 50 to 69, they are 9%–14% higher among men. At the highest ages, the pattern reverses again, with women having slightly more contacts.

The aim of our study is to model COVID-19 transmission taking into account the two crucial demographic factors age and sex. We develop an SEIRD-model that incorporates age- and gender-specific contacts, which shape transmission rates. The model may be used for short- and long-term projections, our example explores short-term effects up to two and a half months of hypothetical changes in contact rates and is restricted to early phases of the pandemic when only non-pharmaceutical mitigation measures (NPMM) are available and no vaccination has been developed. The model can be used to develop scenarios which address the effects of age- and gender-specific changes in contacts due e.g. to the closing of schools, kindergarten and shops, or work in home office, as well as to explore the effect of lifting these measures. However, we use the model to show how gender-specific contacts are associated with infections and deaths. We developed four scenarios which are based at the end of a hypothetical lockdown and set in after the incidence rate has declined to the magnitude called for in [17], which is defined as 10 new cases per million per day or, equivalently, 830 new infections per day in Germany. The first scenario reflects a continuation of the lockdown; the second assumes a lifting of measures mainly at working ages, and the third extends this to children, adolescents, and young adults. In the fourth scenario, contact rates of women are hypothetically aligned to those of men.

The manuscript is structured as follows: First we introduce the basic SEIRD model and discuss how age- and sex-specific contact modelling was incorporated. We present the numerical implementation of the model, model fitting and the development of uncertainty intervals. Then we introduce our scenarios and present the projection results in terms of number of active infections (prevalence), and cumulated number of deaths. We also explore how increasing contacts affect sex ratios in infections and deaths. We close with a discussion of the results, the strengths and limitations of our model, as well as policy implications.

## Materials and methods

**Fig 3:**
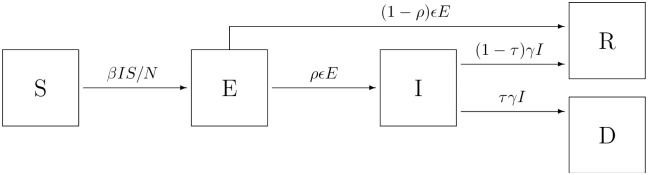
SEIRD compartment model for COVID-19. SEIRD compartment model with 5 transitions. (*S* → *E*: susceptible person becomes exposed to the virus, *E* → *I*: exposed person becomes infectious, *E* → *R*: exposed person is removed due to recovery, *I* → *R*: infectious person is removed due to recovery, *I* → *D*: infectious person is removed due to death).

The core of the epidemiological model is an SEIRD compartment model (see [18]) consisting of the epidemiological states *S* (susceptible, i.e. not yet exposed to the virus), *E* (exposed, but not infectious), *I* (infectious), *R* (recovered), and *D* (dead). The compartments represent individual states with respect to contagious diseases, i.e. COVID-19 in this case, and the transitions between them are considered on a population level (see Fig 3). In this sense, the compartment model is used to describe a population process, but is not intended to model individual processes with respect to COVID-19.

The following essential rate and fraction parameters are involved in the model:

- *β* (contact rate): the average number of individual contacts per specified timespan that are potentially sufficient to transmit the virus (see below for detailed specification)
- *ρ* (manifestation index, fraction): the fraction of people who become infectious at some time after being exposed to the virus
- *ϵ* (incubation rate): the mean rate of exposed people to become infectious; 1/*ϵ* is the average incubation time
- *γ* (recovery rate): the mean rate of exiting the infectious state, either to recovery or death; 1/*γ* is the average duration of the disease
- *τ* (infection fatality rate): the fraction of people who die due to COVID-19

We provide a public repository [19], which contains the dataset and the implementation used for all analyses.

### Contact modeling

The contact model is considered for a population of *N* individuals, which is decomposed into *A* disjoint groups. For each group *a* = 1, …, *A*, the proportion of individuals with regard to the whole population is *N*_*a*_/*N*, where *N*_*a*_ denotes the number of individuals in group *a*. For any *a* ∈ {1,.., *A*} and *b* ∈ {1, …, *A*}, let λ_*ab*_ be the average number of contacts of an arbitrary individual from group *a* with individuals in group *b* during a fixed base time unit *δ*, e.g. 24 hours.

More specifically, define *η*_*ab*_(*t*_1_, *t*_2_) as the random number of contacts of an individual in group *a* with any individual from group *b* over the timespan [*t*_1_, *t*_2_] and $\eta_{a*}\left(t_{1},t_{2}\right):=\sum_{b=1}^{A}\eta_{ab}\left(t_{1},t_{2}\right)$ as the (random) overall number of contacts of an individual from group *a*. It is assumed that *η*_*ab*_(*t*_1_, *t*_2_) is Poisson distributed as

$$\begin{array}{c} \eta_{ab}\left(t_{1},t_{2}\right)∼\text{Poi}(\int_{t_{1}}^{t_{2}}\mu_{ab}\left(s\right)\;ds) \end{array}$$ (1)

via the contact intensity *μ*_*ab*_(*t*). By assuming independence of contacts to different groups, it follows that *η*_a*_(*t*_1_, *t*_2_) is also Poisson distributed having intensity $\mu_{a*}\left(t\right)=\sum_{b=1}^{A}\mu_{ab}\left(t\right)$. The average rate of contact of any individual from group *a* with group *b* is then obtained as

$$\begin{array}{c} \lambda_{ab}≔\int_{0}^{\tau }\mu_{ab}\left(s\right)\;ds, \end{array}$$ (2)

where for the sake of simplicity we assume that *μ*_*ab*_(*t*) is periodic in the sense that *μ*_*ab*_(*t* + *δ*) = *μ*_*ab*_(*t*) for all *t* ≥ 0. Deviations from these assumptions can be incorporated by appropriate modifications to the contact model and parameter set. In the compartment modeling approach, individuals within each group are generally assumed to be homogenous with respect to contact behaviour and no individual effects are considered.

### Group-specific system of ODEs

In order to address the potential impact of the implementation and easing of lockdown measures, we expand the model structure to group-specific compartments. Below, we define groups according to sex and age group, but the following reasoning is valid for any specification of disjoint groups, given that the resulting groups are sufficiently large. Specifically, for given groups *a* = 1, …, *A* and any time *t*, set *S*_*a*_(*t*) as the number of susceptible people in group *a* at time *t*, *E*_*a*_(*t*) as the number of exposed people in group *a* at time *t*, and so on. The group-specific compartment model is characterised by the ODE system

$$\begin{array}{ccc} dS_{a}/dt & = & -\sum_{b=1}^{A}\beta_{ab}I_{b}S_{a}/N_{b}\\ dE_{a}/dt & = & \sum_{b=1}^{A}\beta_{ab}I_{b}S_{a}/N_{b}-\epsilon E_{a}\\ dI_{a}/dt & = & \rho \epsilon E_{a}-\gamma I_{a}\\ dR_{a}/dt & = & \left(1-\rho \right)\epsilon E_{a}+\left(1-\tau_{a}\right)\gamma I_{a}\\ dD_{a}/dt & = & \tau_{a}\gamma I_{a} \end{array}$$ (3)

for all groups *a* = 1, …, *A*, which is a direct extension of the ODE system of the basic compartment model for the special case *A* = 1. We define

$$\begin{array}{c} \beta_{ab}=w\left(1-m_{ab}\right)\left(1-r\right)\left(1-h_{b}\right)\lambda_{ab} \end{array}$$ (4)

as the effective contact rate between groups *a* and *b*, where *w* is the secondary attack rate, *m*_*ab*_ is the specific mitigation effect by lockdown measures with regard to contacts between groups *a* and *b*, *r* is a general factor that accounts for compliance to distance, isolation and quarantine orders, *h*_*b*_ is the proportion of infectious people in group *b* in need of hospitalisation and λ_*ab*_ is the basic contact rate between groups *a* and *b* when no lockdown measures are in place. As we are primarily interested in short-term prediction, we do not model biological aging, i.e. transitions between demographic groups. Therefore, for any time *t*, compartment-specific additivity is assumed, i.e. *S*(*t*) = ∑_*a*_ *S*_*a*_(*t*), *E*(*t*) = ∑_*a*_ *E*_*a*_(*t*), *I*(*t*) = ∑_*a*_ *I*_*a*_(*t*), *R*(*t*) = ∑_*a*_ *R*_*a*_(*t*) and *D*(*t*) = ∑_*a*_ *D*_*a*_(*t*) and *N* = *S*(*t*) + *E*(*t*) + *I*(*t*) + *R*(*t*) + *D*(*t*). The system is closed, meaning that the sum of all ODEs is 0 at each time *t*.

**Fig 4:**
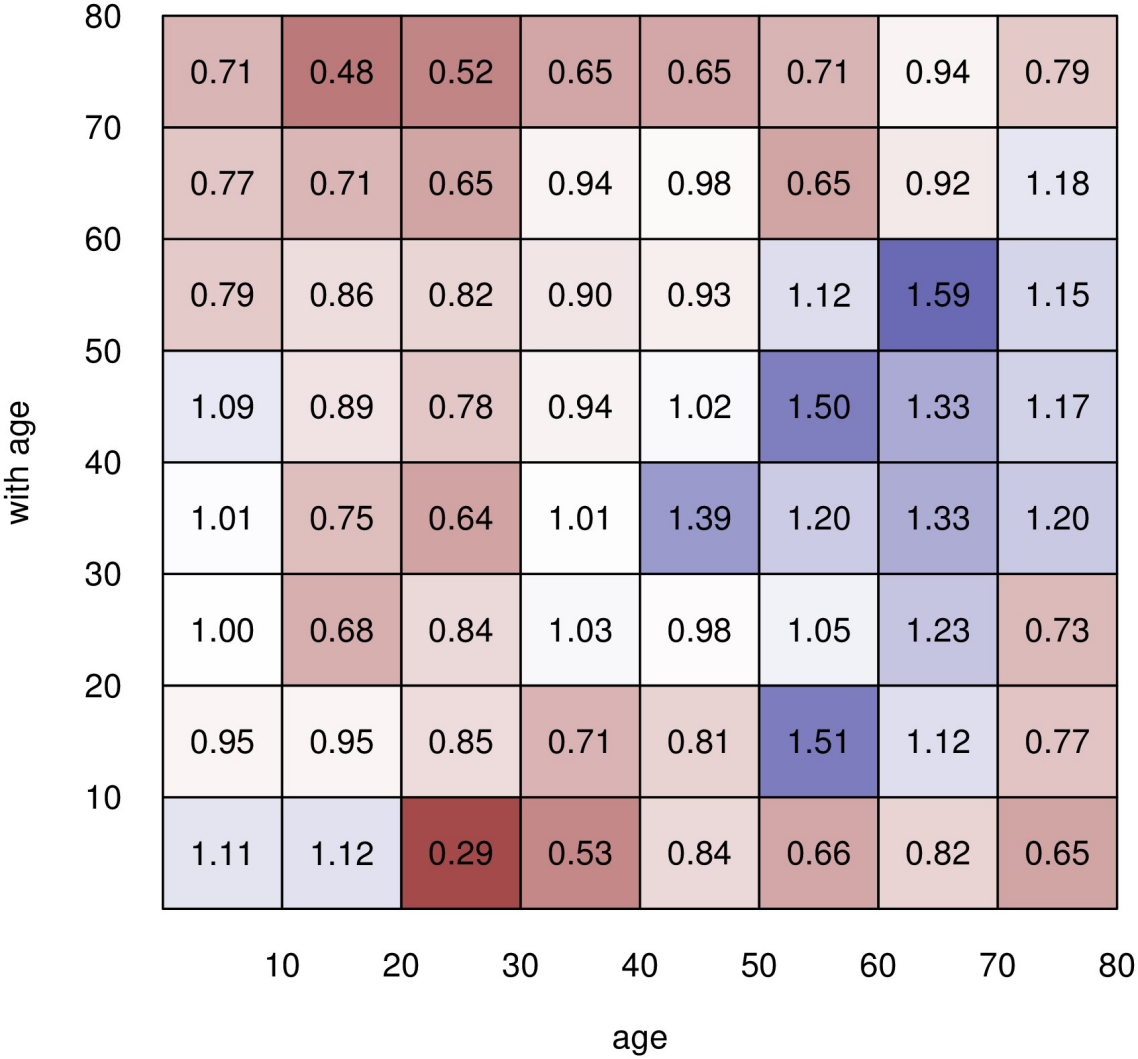
Differences in contact rates by sex. Sex ratios (men/women) of overall contact rates λ_*ab*_ in Germany for different sex and age groups in the absence of lockdown measures (based on [15]).

In the absence of any lockdown measures, the general contact patterns are characterised by the basic contact rates λ_*ab*_, which represent how intensive/often group *a* has any contact with group *b* sufficient for potential virus transmission. In the POLYMOD study [16], 7,290 participants from 8 countries including Germany reported the number and extent of their social contacts during a randomly assigned 24 hour period, using a written diary. The age and gender of the contacted persons were recorded, among other information. Overall, the study contains information on 97, 904 contacts, distributed across the 8 participating countries. For Germany, the matrix of age-specific gender ratios of contact rates is shown in Fig 4. Squares in red color stand for higher contact rates among women (values below one), blue squares for higher contact rates among men (values above one). In 41 of 64 cells (8 × 8 age groups), women have higher contact rates than men (Table 1). This is especially true for the youngest age group, 0–9 years, where contacts with women aged 20 and above are always far higher than contacts with men of these ages. Among adolescents (ages 10–19), there is still a surplus of female contacts, although men dominate contacts with persons aged 50 and older. This pattern is reinforced in the 20–29, 30–39, and 50–59 age groups. Female contacts again predominate in the 60–69 and 70–79 age groups, and this trend becomes stronger with age.

**Table 1:** Sex ratios of overall contact rates in Germany.

Age group	0–9	10–19	20–29	30–39	40–49	50–59	60–69	70–79
0–9	1.11	0.96	1.01	1.01	1.09	0.79	0.77	0.71
10–19	1.12	0.96	0.68	0.74	0.89	0.87	0.71	0.47
20–29	0.29	0.86	0.85	0.64	0.78	0.83	0.65	0.52
30–39	0.53	0.71	1.03	1.01	0.95	0.90	0.95	0.65
40–49	0.85	0.82	0.98	1.40	1.02	0.93	0.98	0.65
50–59	0.66	1.51	1.05	1.21	1.50	1.13	0.65	0.71
60–69	0.83	1.13	1.23	1.32	1.33	1.59	0.92	0.95
70–79	0.65	0.77	0.73	1.21	1.18	1.17	1.10	0.79

Sex ratios (men/women) of overall contact rates λ_*ab*_ in Germany for different sex and age groups in the absence of lockdown measures (based on [15]).

The behaviour of the epidemiological model is primarily governed by the effective contact rates *β*_*ab*_ which result from the basic contact rates λ_*ab*_ by accounting for the secondary attack rate and lockdown measures. It is implicitly assumed here that hospitalised cases are effectively isolated from the remaining population and can not spread the disease. Note that the product (1 − *m*_*ab*_)(1 − *r*)(1 − *h*_*b*_) represents the proportion of potential virus transmissions that are not prevented.

### Numerical implementation

We have implemented the suggested model in R using a discrete approximation of the ODE system via the Forward Euler Method (see [20]). The step size Δ*t* is chosen as a quarter fraction of one day. Accordingly, the transition rates between the compartments need to be adjusted, whereas the fraction parameters remain unchanged. For instance, if the average incubation time is 5 days and Δ*t* = 1/4 (days), the transition parameter *ϵ* = 1/5 ⋅ 1/4 = 1/20, whereas the manifestation index *ρ*, as the relative proportion of exposed people developing symptoms, is the same for any Δ*t*. The time-discrete approximation of the system of ODEs is therefore described as follows.

$$\begin{array}{ccc} \Delta S_{a} & = & -\sum_{b=1}^{A}\beta_{ab}I_{b}S_{a}/N_{b}\Delta t\\ \Delta E_{a} & = & \sum_{b=1}^{A}\beta_{ab}I_{b}S_{a}/N_{b}\Delta t-\epsilon E_{a}\Delta t\\ \Delta I_{a} & = & \rho \epsilon E_{a}\Delta t-\gamma I_{a}\Delta t\\ \Delta R_{a} & = & \left(1-\rho \right)\epsilon E_{a}\Delta t+\left(1-\tau_{a}\right)\gamma I_{a}\Delta t\\ \Delta D_{a} & = & \tau_{a}\gamma I_{a}\Delta t \end{array}$$ (5)

For the involved epidemiological parameters, estimates are available from [21, 22]. [23] provide estimates of the age- and sex-specific infection fatality rates, based on a seroepidemiological study.

### Data

**Fig 5:**
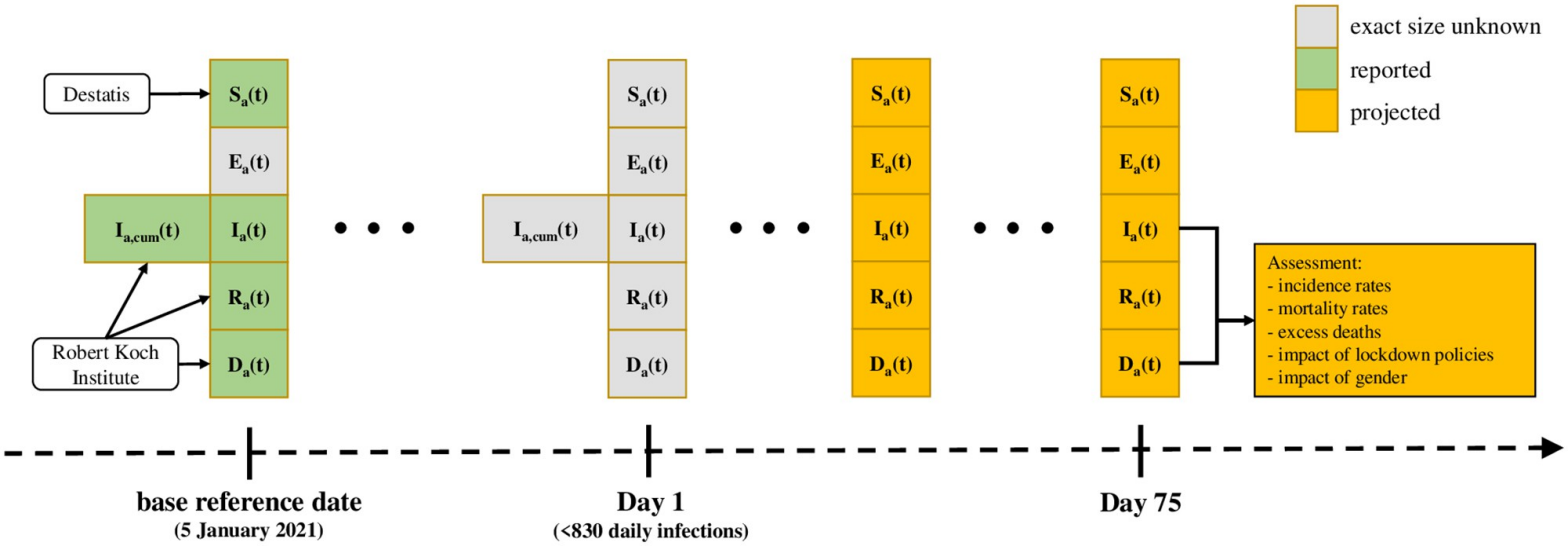
Diagram of the scenario analysis approach. Outline of the scenario analysis. For every compartment *C*, *C*_*a*_(*t*) denotes the number of people from group *a* which are in compartment *C* at time *t*; *I*_*a*,cum_ denotes cumulative number of infections. *S*_*a*_(*t*) on the base reference date are obtained from Destatis (Federal Statistical Office of Germany); *I*_*a*_(*t*), *R*_*a*_(*t*) and *D*_*a*_(*t*) on the base reference date are obtained from the Robert Koch Institute Dashboard.

We use data provided by the Robert Koch Institute (RKI), which is by law (German Infection Protection Act) responsible in Germany to prevent and control epidemic diseases as well as to inform other institutions and the public in epidemics of national scope (Fig 5). As part of this purpose, the RKI established an online dashboard, through which current epidemiological information including the number of notified infections and the individual age and sex characteristics of the infected cases is published daily. These information on infections and case characteristics is obtained through a national epidemiological reporting system, which had been established prior to the pandemic.

Based on the data reported on the dashboard, we have deduced the number of newly reported infections, number of actively infected, number of recoveries, and number of deaths related to COVID-19 for each day from January 1, 2020 to January 5, 2021.

### Model fitting

We suggest to fit the model along the following consecutive steps:

1. Determine a timespan {1, …, *T*} during which no lockdown measures had been in place, and determine the cumulative number of infections during this time.
2. Based on plausible ranges for the involved compartment parameters and the initial state of the compartment model, fit the contact intensity model with regard to the cumulative number of infections during {1, …, *T*}.

In order to derive the secondary attack rate *w* from the contact rates λ_*ab*_ given in [15], we fit the proposed compartment model to the reported cases during a timespan {1, …, *T*} of no lockdown. This step is necessary, because the social contact rates λ_*ab*_ do not incorporate the specific transmission characteristics of SARS-CoV-2, such as the average length of the infectious period and average infection probability per contact. We assume that *w* is not specific to age or sex. We employ

$$\begin{array}{c} Q\left(w\right)≔\sum_{t=1}^{T}{\left({\hat{I}}^{\text{cum}}\left(t|w\right)-I^{\text{cum}}\left(t\right)\right)}^{2} \end{array}$$ (6)

as a least-squares criterion function in order to determine the optimal value $\hat{w}≔{\text{argmin}}_{w>0}Q\left(w\right)$, where *I*^cum^(*t*) are the observed cumulative infections, and ${\hat{I}}^{\text{cum}}\left(t|w\right)$ are the estimated cumulative infections based on the epidemiological model given *w*. Hence, $\hat{w}$ is the scalar parameter for which the cumulative infections are best predicted retrospectively. Note that the observed cumulative number of infections is usually recorded for each day, while the step size Δ*t* in the model may be different. Thus, appropriate matching of observed and estimated values is necessary.

This fitting method requires that the number of infections for the considered geographical region is sufficiently large, such that the mechanics of the compartment model are plausible. Note that potential under-ascertainment may not substantially change the optimal value of *w* as long as the proportion of detected cases does not strongly vary over time. Furthermore, the suggested fitting method is based on the assumption that the probability of virus transmission is independent of age and sex, given that a contact has occurred. If different propensities of virus transmission are allowed for, the contact matrix may be correspondingly adjusted along introduced parameters *w*_1_, …, *w*_*ab*_ for each group combination or *w*_1_, …, *w*_*a*_, if the probability of transmission only depends on the contact group. The criterion function is likewise extended as (*w*_1_, …, *w*_*ab*_) ↦ *Q*(*w*_1_, …, *w*_*ab*_). However, optimisation in this extended model requires a sufficiently large number of transmissions and detailed information on the recorded infections, and may lead to unpractically vague estimates otherwise. Therefore, we employ the simpler model with univariate *w* first.

### Sensitivity analysis and parameter uncertainty

In order to account for parameter uncertainty, we develop uncertainty intervals for the number of people in each compartment. As a cautionary remark, note that these intervals are not to be equated to confidence intervals in the classical sense. Though the resulting intervals are conceptually comparable to Bayesian credibility intervals, they are to be distinguished in that no prior distribution is explicitly assumed here. Note that these intervals do not reflect uncertainty in terms of the underlying infection data.

We predict the number of cases in each age-specific compartment using a Monte Carlo simulation method. For each simulated run, all parameters are independently drawn from their respective range, yielding an instance of a hypothetical parameter setup. Given these parameters, the SEIRD ODE model is approximated using the Forward Euler Method and known initial states, as described above. After *N*_*R*_ of such simulated runs, the prediction intervals for all relevant values are construed based on the pseudo-empirical trajectories of the compartment model. Furthermore, prediction intervals are derived as point-wise quantile ranges for each *t*. For instance, an 80% prediction interval for the number of infectious people in group *a* at time *t* is [*I*_*a*,10%_(*t*), *I*_*a*,90%_(*t*)].

### Analytical approach and scenarios

First, we fitted the model to observed COVID-19 infections using transition rates from literature for the period February 21 to March 13, 2020, where no lockdown measures were present. We estimated the model parameter *w*, also termed secondary attack rate, which reflects the probability of infection per contact, by least squares estimation with regard to observed and predicted values, as described above.

Second, we developed four scenarios starting our projections on the hypothetical day, when the incidence rate during the lockdown has declined to the magnitude called for in [17], which is defined as 10 new cases per million per day or, equivalently, 830 new infections per day in Germany. In a separate step, we estimate that at this point the cumulated number of infections (∑_*a*_ *I*_*a*,*cum*_(*t*), see Fig 5) is about 3 million. The sizes of all compartments are accordingly adjusted.

We consider 75 days for our projections and use quarter-days as the base time step length Δ*t*. In Scenario 1, which can be considered as a baseline scenario, we assume that the age- and sex-specific contacts are reduced by 80%, i.e. only 20% of the contacts estimated by [15] are realized between start and end of the projection. This applies to all age groups and to both sexes. This scenario should reflect permanent distancing measures as are in force on January 5, 2021. Scenario 2 assumes that contacts at working ages 30–59 were increased by 5 percentage points (PP), and among those aged 60–69 by 2.5 PP, equaling a decline of 76% and 78% respectively. All other ages remain at 80% contact reduction. This should reflect the return from home office settings, the opening of shops, cafes and restaurants. Scenario 3 considers an additional increase in contact rates among ages 10–29 by 5 PP, which should reflect the opening of schools and venues mainly visited by young individuals. Scenario 4 explores the impact of sex-specific contacts by aligning the female contacts to the level of male contacts. We explore the following age-specific outcomes:

1. Number of active infections which were defined as the number of individuals in compartment *I* by age and sex,
2. Cumulative number of deaths out of compartment *I* by age and sex,
3. Excess number of deaths in Scenarios 2, 3 and 4 in comparison to Scenario 1 by age and sex,
4. Sex ratio of incidence defined as men/women ratio of the number of new COVID-19 cases divided by the total population section,
5. Sex ratio of mortality rate defined as men/women ratio of the number of deaths out of compartment *I* divided by the total population section.

## Results

**Fig 6:**
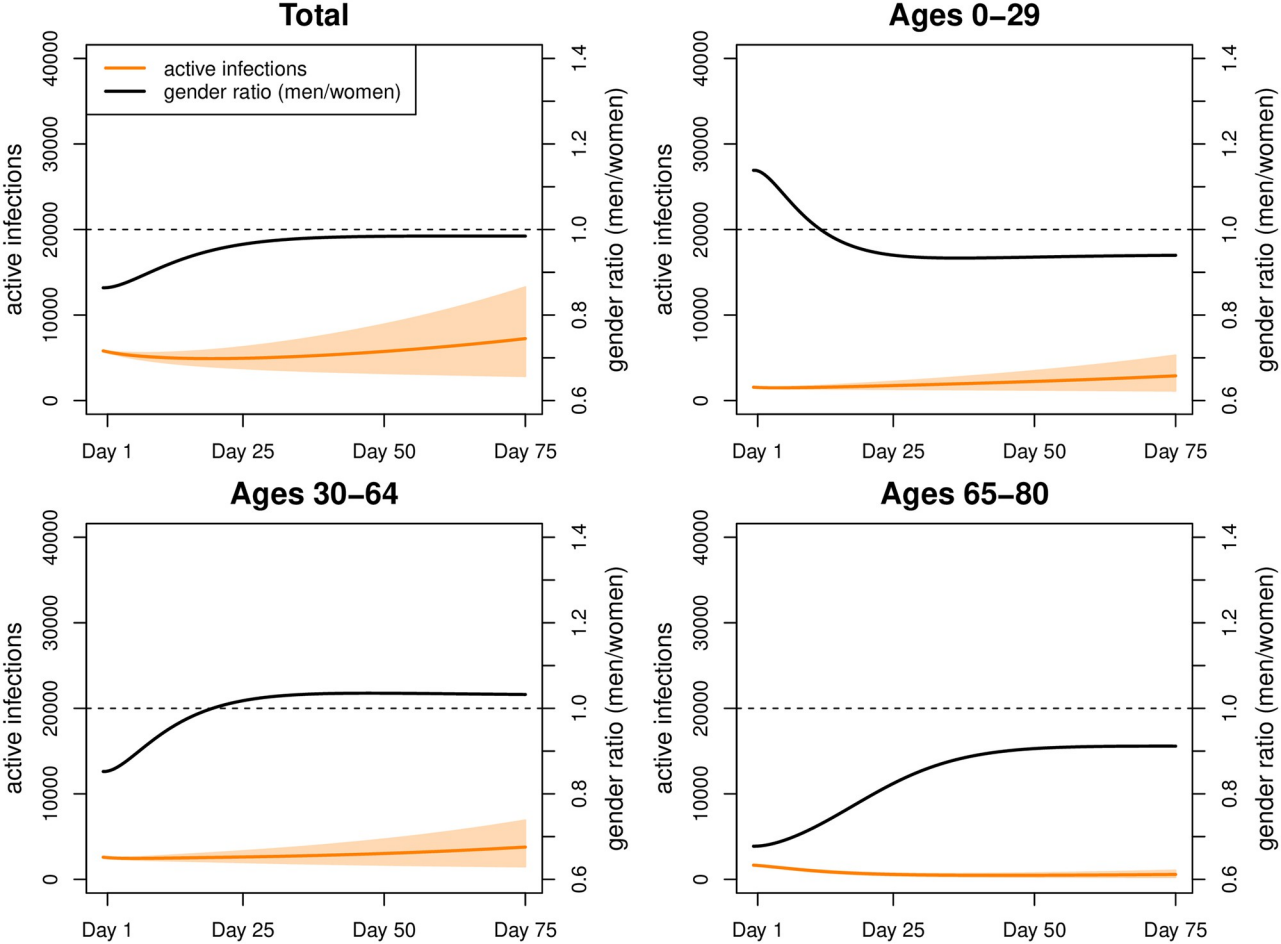
Simulation results for Scenario 1. Number of active infections and gender ratio (men/women) in Scenario 1 (intervals represent 80% range due to parameter uncertainty).

Fitting our model to COVID-19 infections observed during our fitting period (February 21—March 31, 2020) results in an estimate of the secondary attack rate *w* ≈ 13%. We started with 5,810 active infections on day 1, reflecting the intended overall incidence rate of 10 new infections per million people per day. Under Scenario 1, this figure increased to approximately 7,190 (Fig 6) (men: 3,567; women: 3,633) by day 75. The number of active infections was highest at ages 30–39 (men: 613; women: 673), followed by ages 10–19 (men: 589; women: 624), and ages 40–49 (men: 581; women: 572). The cumulative number of deaths increased from 65,792 to 66,133 with 34,576 men and 31,557 women. By day 75, infection rates (Table 2) were highest among the 10–19-year old (men 17.2 and women 15.3 per 1000 individuals) followed by ages 30 to 49 (above 11 for both genders), and ages 0–9 (around 10 for both genders). At ages above 50, infection rates declined rapidly, almost halving from individuals in their fifties (men: 8.7; women: 7.4) to those in their sixties (men: 5.0; women: 4.1), while at older ages the decline followed at a much lower pace (ages 70–79: men: 2.8; women: 3.0; ages 80+: men: 2.2; women: 1.9). Sex ratios of infections were below 1 in the age interval 10 to 49, indicating a higher risk of infections among women. From age 50 onwards they were generally above 1 (with the exception of ages 70–79), thus turning the disadvantage towards men. As expected, death rates (Table 3) increased exponentially with age. They were more than twice to three times as high among men than women.

**Table 2:** Mean infection rates (in %) and male/female ratio.

Mean infection rates (in %)										
		0–9	10–19	20–29	30–39	40–49	50–59	60–69	70–79	80+
Scenario 1	female	0.0109	0.0172	0.0098	0.0128	0.0115	0.0074	0.0041	0.0030	0.0019
	male	0.0114	0.0153	0.0071	0.0110	0.0115	0.0087	0.0050	0.0028	0.0022
	ratio	1.0459	0.8920	0.7267	0.8613	0.9998	1.1843	1.2066	0.9363	1.1872
Scenario 2	female	0.0150	0.0208	0.0178	0.0299	0.0211	0.0168	0.0067	0.0031	0.0019
	male	0.0122	0.0117	0.0077	0.0198	0.0195	0.0252	0.0122	0.0038	0.0032
	ratio	0.8100	0.5600	0.4300	0.6600	0.9200	1.5000	1.8200	1.2500	1.6800
Scenario 3	female	0.0205	0.0420	0.0301	0.0424	0.0312	0.0244	0.0093	0.0044	0.0028
	male	0.0166	0.0221	0.0130	0.0279	0.0283	0.0369	0.0169	0.0054	0.0046
	ratio	0.8100	0.5300	0.4300	0.6600	0.9100	1.5100	1.8200	1.2300	1.6400
Scenario 4	female	0.0168	0.0324	0.0212	0.0354	0.0276	0.0231	0.0083	0.0029	0.0021
	male	0.0144	0.0189	0.0111	0.0244	0.0248	0.0324	0.0149	0.0047	0.0040
	ratio	0.8600	0.5800	0.5200	0.6900	0.9000	1.4000	1.7900	1.6100	1.9000

Mean infection rates (in %) and male/female ratio. The infection rates are determined with respect to projected active infections after 75 days.

**Table 3:** Mean death rates (in %) and male/female ratio.

Mean death rates (in %)										
		0–9	10–19	20–29	30–39	40–49	50–59	60–69	70–79	80+
Scenario 1	female	0.0000	0.0001	0.0000	0.0001	0.0002	0.0006	0.0010	0.0035	0.0091
	male	0.0001	0.0000	0.0000	0.0000	0.0003	0.0013	0.0033	0.0071	0.0237
	ratio	–	–	0.4100	0.4000	1.8700	2.2700	3.2400	2.0500	2.5900
Scenario 2	female	0.0000	0.0000	0.0000	0.0002	0.0003	0.0007	0.0016	0.0028	0.0084
	male	0.0001	0.0000	0.0000	0.0001	0.0007	0.0017	0.0053	0.0059	0.0257
	ratio	–	–	0.4200	0.5100	2.0500	2.6400	3.2400	2.1500	3.0600
Scenario 3	female	0.0000	0.0001	0.0001	0.0002	0.0004	0.0008	0.0019	0.0031	0.0091
	male	0.0001	0.0000	0.0000	0.0001	0.0008	0.0021	0.0061	0.0067	0.0282
	ratio	–	–	0.4100	0.5000	2.0300	2.6800	3.2600	2.1300	3.0900
Scenario 4	female	0.0000	0.0001	0.0001	0.0002	0.0004	0.0008	0.0018	0.0025	0.0083
	male	0.0001	0.0000	0.0000	0.0001	0.0008	0.0020	0.0058	0.0063	0.0272
	ratio	–	–	0.4800	0.5200	2.0100	2.4800	3.1900	2.5800	3.2500

Mean death rates (in %) and male/female ratio. The death rates are determined with respect to projected active infections after 75 days and scaled to annual rates.

**Fig 7:**
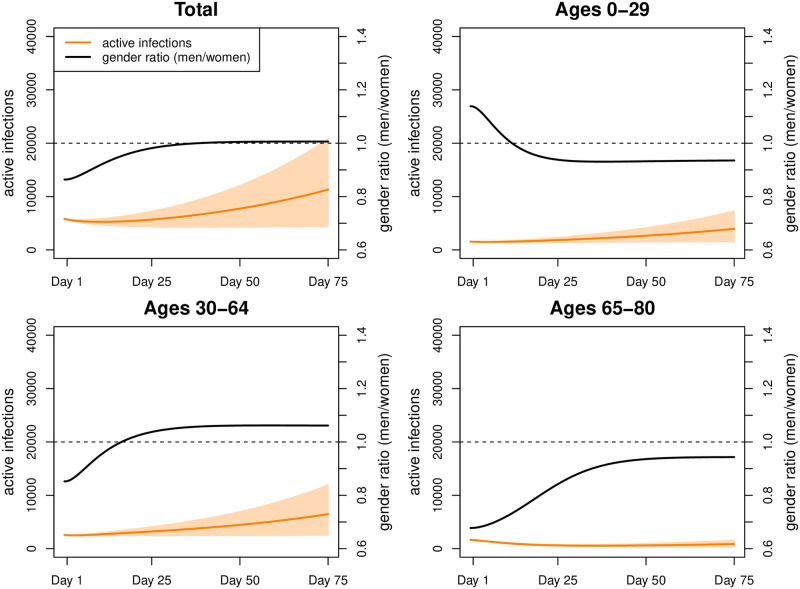
Simulation results for Scenario 2. Number of active infections and gender ratio (men/women) in Scenario 2 (intervals represent 80% range due to parameter uncertainty).

Scenario 2 assumed increased contacts at working ages and arrived at 11,178 active infections by day 75 (Fig 7) and therefore 3,988 active infections more than in Scenario 1 (men 2,039; women 1,949). These additional infections stemmed from all ages, even if the risk of infections increased most among the working ages. Sex ratios of infection rates turned toward the disadvantage of men from age 50 onwards.

The additional infections translated into an additional 50 deaths (Table 4; men: 34; women: 16); among women, 54% of these deaths resulted at ages 70 and above; among men, 53%, reflecting their higher mortality already at younger ages. Also the gender ratios of death rates turned towards the disadvantage of men.

**Table 4:** Mean excess number of deaths.

		0–9	10–19	20–29	30–39	40–49	50–59	60–69	70–79	80+	Total
Scenario 2	female	0	0	0	1	1	2	3	4	5	16
	male	0	0	0	0	1	6	9	8	10	34
Scenario 3	female	0	0	0	1	1	4	5	8	11	30
	male	0	0	0	0	2	10	15	16	22	65
Scenario 4	female	0	0	0	1	1	4	4	0	4	14
	male	0	0	0	0	2	9	12	12	17	52

Mean excess number of deaths in Scenarios 2–4 in comparison to Scenario 1.

**Fig 8:**
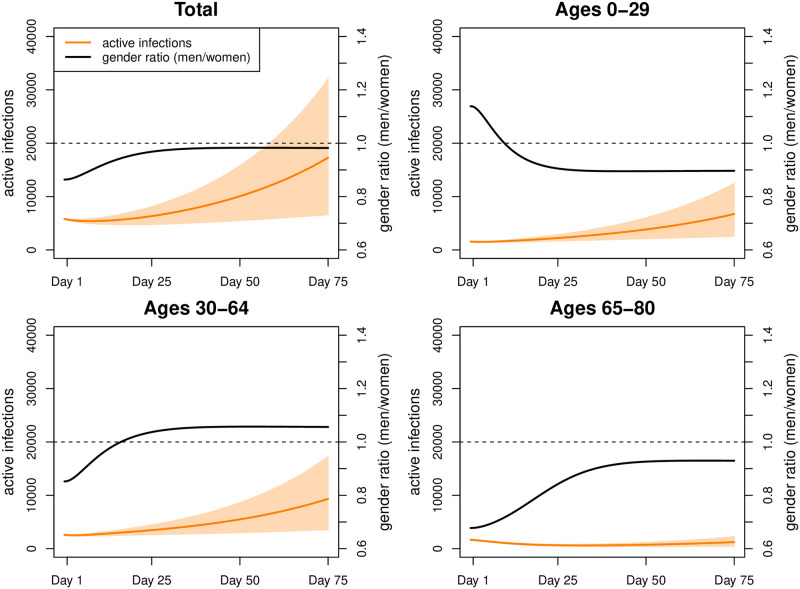
Simulation results for Scenario 3. Number of active infections and gender ratio (men/women) in Scenario 3 (intervals represent 80% range due to parameter uncertainty).

Scenario 3 with increased contacts at young and working ages resulted in 17, 001 active infections (Fig 8) and thus 9, 812 more than in Scenario 1 (men: 4, 857 women: 4, 955) which translated into an additional 95 deaths (Table 4) with the majority resulting from ages 70 and above (men: 58%; women: 63%). Sex ratios, both in infections and deaths, only changed marginally as compared to Scenario 2.

**Fig 9:**
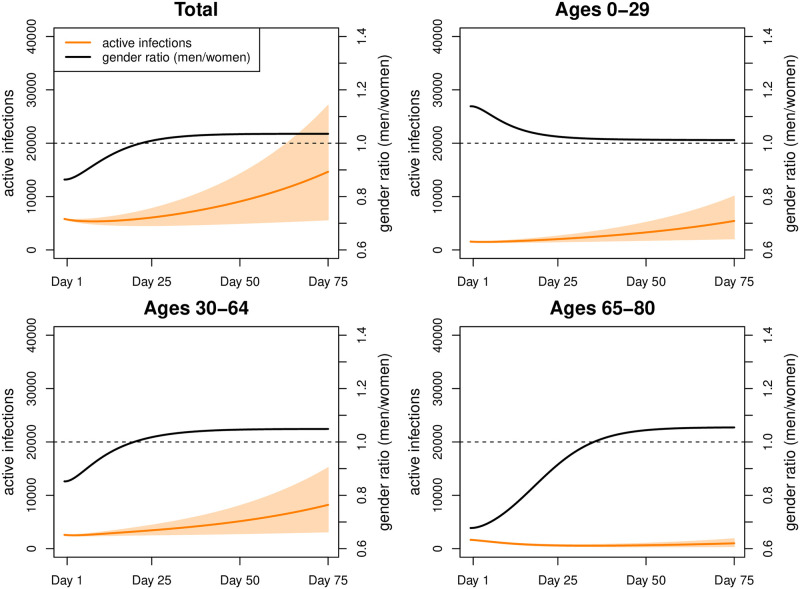
Simulation results for Scenario 4. Number of active infections and gender ratio (men/women) in Scenario 4 (intervals represent 80% range due to parameter uncertainty).

Scenario 4 used similar assumptions as Scenario 3 but the contact rates of women were lowered to those of men. This translated into 14, 434 active infections (Fig 9) which are 7, 244 more than in Scenario 1, but 2, 567 less than in Scenario 3. More infections were spared among women (−1, 485) than among men (−1, 082). While the number of excess deaths (Table 4) was still higher than in Scenario 1 (men: 52; women 14), it was lower than in Scenario 3 (men: 65−52 = 13; women: 30−14 = 16). Thus, in absolute terms, men profited almost as much as women from reduced contacts among women.

## Discussion

Incorporating age- and gender-specific contact rates in a COVID-19 compartment model permits exploration of the effects of changes in mitigation measures on the two genders. We developed four scenarios which assumed ongoing distancing measures versus easing of contact restrictions in working ages, and among adolescents and young adults. Our projections do not set out to forecast the actual number of COVID-19 infections in a time span of about two and a half months, they rather assess the effect of increased contacts on the infection and mortality risks of the two genders and the various age groups.

Three important lessons can be learned from our scenarios.

First, even a small change in contact rates has a large impact on infections and deaths. In our projections we assumed an increase ranging from 2.5 to 5 PP, resulting in an increase in infections of between 50% and 236%. This reflects the fact that without NPMM such as masks, physical distance between individuals, better air ventilation and hygiene, and without contact tracing, the infection rates would return to the initial exponential increase. This was reflected in a reproduction rate of 3.3 to 3.8, as observed at the beginning of the pandemic ([24–27]). However, the presence of NPMM also mitigates the effect of the increase in contacts due to the return to office, opening of shops, restaurants, as well as schools, and venues visited by young adults, leaving it far from the initial impact. In our present scenarios, both effects, the change of contact rates and the change of their impact, are captured in the reduction matrix (*m*_*ab*_), which is multiplied with the matrix of the contact rates. One alternative approach would be to develop separate scenarios for changes in the secondary attack rate *w* due to NPMM and changes in the contact rates (*m*_*ab*_), which is one possibility to modify this analysis further. At any rate, our scenarios show that small changes already have large impacts on infections and deaths. This implies that the impact of contacts must be diminished considerably to allow increases in contacts without returning to exponential growth of infections, hence underlining the high importance of the NPMM in the current phase of the pandemic.

Second, due to intergenerational contacts, any easing of measures in working and young ages will inevitably lead to an increase in infections and deaths at all ages. People at old ages will suffer most with elderly men being at a particular high risk of death due to increased contacts. Most interestingly, this increased mortality is also transmitted by the higher contact rates of women, as shown in our Scenario 4. Mortality may have changed over the course of the pandemic because of better treatment options of critically severe COVID-19 cases using, e.g., dexamethasone [28]. Our mortality rates based on [23] are based on Spanish data from April 27—June 22, 2020, which already should reflect a possible decline. Our results emphasise that increases in contacts need to be accompanied by special measures protecting the elderly from death, without negative physical and mental health consequences due to quarantine and isolation measures [29]. Contrary to deaths, infections will mainly increase at young and middle ages with a lower risk of severe COVID-19 symptoms or even asymptotic disease courses.

Third, small increases in contact rates change the gender ratios in infections and deaths towards the disadvantage of men. At all ages, men will have more than twice the mortality risk from COVID-19, while the risk of infections is more frequent among working age women than men. At old ages, men have higher infection risk. Note that, in absolute numbers, more women are diagnosed with COVID-19 at old age due to their higher life expectancy. Here a more substantial question arises, namely whether COVID-19 infection rates are indeed gender-specific. German COVID-19 infection rates, as in any other country, are biased by the time-lag of reporting and by differential availability of polymerase chain reaction (PCR) tests over time and to subgroups of the population [27]. The gender-specific diagnoses in favor of women may reflect the higher contact intensity of women in general and their occupation in health and care professions, which may have led to a higher rate of PCR tests performed and thus a lower number of undiagnosed cases. In addition, women are more health conscious than men [30]. They are not only more adherent to NPMM [31], but may also have utilized PCR testing at a higher rate even when symptoms were less severe. While there is a general lack of information in relation to COVID-19, there is evidence of gender patterns in health information seeking, with women performing better than men [32]. Men also tend to underestimate their health risks, which may lead them to avoid risk information messages [33]. On the other hand, [13] found sex-specific differences in immune response to COVID-19 infections. For a further discussion of potential sex-specific mechanisms modulating the course of disease, see also [34]. Thus, we can conclude that both biological and social factors contribute to sex- and gender-specific infection and mortality rates.

A sizeable proportion of infections and deaths is transmitted through the higher contact rates of women, as shown in our Scenario 4. This higher number of contacts may primarily result from care obligations where women are the main care providers. By mid-July, among the COVID-19 infection cases reportedly cared for or working in medical facilities, 72% were women and 28% men with a median age of 41 years [35]. Since women have a higher untapped work-from-home capacity than men [36] better exploitation of their work-from-home potential may safe infections and lives.

Our study has a series of strengths and limitations. The fit of our model to the baseline period in February and March results in an estimated secondary attack rate *w* ≈ 13%, putting our findings in close agreement with the rates reported in Guangzhou (China), where the household *w* varied between 12% and 17%, and the non-household *w* between 6% and 9% [37], although higher attack rates of up to 35% have been reported e.g. for meals and holiday visits [38]. Thus, we can conclude that our model reflects well the dynamics of the spread of the infections. We focused on the practical emulation of the dynamic behaviour and process of the spreading of COVID-19 while incorporating specific epidemiological information on the virus and disease. To achieve this aim we used a compartment modeling framework, which has become a standard approach in epidemiology due to its flexibility and accessibility. The main advantage of this modeling framework is that a considerable amount of demographic and epidemiological information can be incorporated while the essential model structure and implementation remain relatively simple. Similarly, it is possible to extend the model to incorporate parameter uncertainty, as described above. Furthermore, we want to emphasize the Markov-like property of compartment modeling in the sense that current compartment sizes on a specific date are sufficient for deducing the subsequent behaviour of the epidemiological process, which makes the framework particularly attractive for forecasting and investigating hypothetical scenarios. However, there is one drawback to compartment modelling that it is inherently based on an averaging rationale which treats population groups homogenously and the average number of contacts in each group is a determining parameter. In contrast to truly stochastic models (such as agent-based models), no random or systematic individual deviations from the fundamental contact patterns are taken into consideration. Likewise, compartment modeling is not suitable for assessing local dynamic behaviour, such as the notions of infection clusters and superspreading events. In addition, geographical and spatial information are not explicitly considered in compartment modeling, and this further limits the scope of the forecasting results.

In general, assessing the impact of introducing or easing different lockdown measures is remarkably difficult, especially because several aspects are usually changed simultaneously and the general behaviour of the population may change dynamically at the same time. Some efforts have been made to address these issues in the literature, however we advise against using the proposed model for such purposes. One main reason is that the initial state for forecasting and fitting of the model relies primarily on available data sources, which are in the form of reported count data. In addition to the general limited validity of observational data, there is still insufficient knowledge on the specific characteristics of COVID-19 and the actual current spread of the virus. Naturally, other modeling approaches face the same issues of data quality.

Data quality is also an issue when it comes to contact rates. Contact behavior may have changed significantly since the 2008 POLYMOD study [16], and it is questionable whether it will ever be the same compared with the pre-COVID-19-pandemic in terms of contacts beyond one’s home and personal life. Future studies need to take a strong gender perspective on how, for example, different work policies resulting in different task allocation [39, 40], changes in NPMM [31], and acceptance of COVID-19 vaccination [41–44] influence and interact with contact behavior.

In our COVID-19 forecasts, the number of infections and the number of deaths differ only slightly from models which do not differentiate by sex or gender. However, age- and gender-specific models based on contact rates provide better insight into populations at risk for infection. A gender-specific understanding of infection pathways is another important aspect of preventing serious clinical outcomes in both men and women. The focus of our model is on the gender aspect in the early phases of the pandemic, when only NPMM are available and no vaccine has yet been developed. Thus, our current model helps to understand the infection pathways in emerging viruses. Since the completion of our model, new variants of SARS-CoV-2 have emerged and vaccination has been successfully introduced. Future models of the COVID-19 pandemic should incorporate these new developments without forgetting the importance of gender in understanding transmission routes.

## Conclusion

Our study results help target public health interventions when resources are scarce, such as who should be tested and vaccinated first for emerging viruses. With respect to COVID-19, gender is associated with infection rates and their consequences, and gender-specific contact rates can model this association. Gender-specific modes of transmission must be considered in all policy decisions. Therefore, to combat SARS-COV-2 and other emerging viruses, we urgently need up-to-date and gender-specific data on contact patterns, infections and deaths.
